# Propensity-score-matched comparison of safety, efficacy, and outcome of intravascular lithotripsy versus high-pressure PTCA in coronary calcified lesions

**DOI:** 10.1016/j.ijcha.2021.100900

**Published:** 2021-12-09

**Authors:** Adem Aksoy, Vedat Tiyerili, Nora Jansen, Muntadher Al Zaidi, Maximillian Thiessen, Alexander Sedaghat, Marc Ulrich Becher, Felix Jansen, Georg Nickenig, Sebastian Zimmer

**Affiliations:** a Heart Center Bonn, Rheinische Friedrich-Wilhelms-Universität Bonn, Germany; b Department of Computer Science, University of Bonn, Germany; c Fraunhofer Institute for Intelligent Analysis and Information Systems, Sankt Augustin, Germany

**Keywords:** Calcification, High-pressure PTCA, Lithotripsy, Shockwave, AMI, Acute myocardial infarction, atm, Atmosphere, CAD, Coronary artery disease, DES, Drug eluting stent, IVL, Intravascular lithotripsy, LAD, Left anterior descending artery, MACE, Major adverse cardiovascular event, MLD, Minimal lumen diameter, NC, non-compliant, PCI, Percutaneous coronary intervention, PSM, Propensity-score-matched, PTCA, Percutaneous transluminal coronary angioplasty, QCA, Quantitative Coronary Analysis, RA, Rotational atherectomy (RA), RCA, Right coronary artery, TIMI, Thrombolysis in myocardial infarction

## Abstract

**Background:**

Data regarding safety, efficacy, and outcome of intravascular lithotripsy (IVL) in comparison to standard techniques are lacking. This study sought to compare IVL with non-compliant high-pressure balloon percutaneous coronary angioplasty (PTCA).

**Methods and results:**

We performed a retrospective propensity-score-matched study to compare procedural success in 57 consecutive patients who received IVL-guided PCI in calcified coronary lesions with 171 matched patients who were treated with high-pressure PTCA with a non-compliant (NC)-balloon. The mean minimal lumen diameter (MLD) for the IVL group was 1.08 ± 0.51 mm, and the median percent diameter stenosis on quantitative angiography was 70.2% (interquartile range, 60.2–78.6%). MLD in the high-pressure dilatation group was 0.97 ± 0.43 mm, and the median percent diameter stenosis was 71.5% (interquartile range, 58.5–77.0%). IVL-guided PCI reduced median stenosis to 17.5% (interquartile range, 9.3–19.8%) with an acute gain of 0.93 ± 0.7 mm. High-pressure dilatation resulted in a final median stenosis of 19.3% (interquartile range, 13.33–28.5%). Procedural success was significantly higher (82.5% vs. 61.4%; p: 0.0035) in the IVL group. MACE through 12 months occurred in 10.5% of cases in the IVL group and in 11.1% of the high-pressure group (p = 0.22). Angiographic complications (coronary dissection, slow or no reflow, new coronary thrombus formation, abrupt vessel closure) were very low (0.2% vs. 0.12%).

**Conclusion:**

IVL resulted in a significantly higher rate of procedural success compared to high- pressure NC-balloon dilatation in patients with calcified coronary lesions. The rate of MACE through 12 months was similar to the standard therapy.

## Highlights

• Calcified coronary lesions are frequently in patients with coronary artery disease.
• Intravascular lithotripsy was shown to be safe and effective for treating calcified lesions in coronary artery disease.
• Data of intravascular lithotripsy in comparison to standard techniques are lacking.
• Intravascular lithotripsy showed higher rate of procedural success without differences in rate of MACE after 12 months in comparison to high- pressure NC-Balloon PTCA.

## 1 Introduction

Calcified coronary lesions are increasingly observed in patients with coronary artery disease (CAD) [1]. Up to 20% of patients who need percutaneous coronary intervention (PCI) present with moderate to severe coronary calcifications. The severity of the calcification present affects significantly the interventional procedural success, survival, myocardial infarction rates, and the rate of target lesion revascularization [2], [3], [4]. Preparation of the lesion by using high-pressure dilatation, scoring/cutting balloons, or rotational atherectomy (RA) devices before stent implantation are recommended to prevent stent failure [5], [6], [7], [8], [9], [10], [11]. However, the above-mentioned strategies are often complicated by vessel dissection, perforation, and vessel occlusion [12]. Due to a high risk of restenosis and stent thrombosis, stent implantation in an incompletely dilated lesion is strictly prohibited. Recently, intravascular lithotripsy (IVL) was shown to be safe and effective for treating calcified lesions in coronary artery disease [13], [14], [15]. The principle of IVL is based on electrohydraulic-generated sonic pressure waves, which are able to disrupt sub-endothelial calcification in the vessel wall [15]. However, clinical data regarding the use of IVL are still scarce. Therefore, the present study sought to compare the preparation of lesions using IVL and high-pressure PTCA to evaluate the safety, efficacy, and outcome of this tool.

## 2 Methods

### 2.1 Patient selection and study design

We conducted a registry study of consecutive patients with calcified coronary lesions who had undergone primary IVL prior to stent implantation from February 2018 to May 2019 at the heart center of the University Hospital in Bonn/ Germany.

The comparison group consisted of a cohort of patients with calcified coronary lesions who were treated with high-pressure non-compliant (NC) balloon dilatation in 2017. To identify the high-pressure PTCA group, all patients who underwent PCI at the Heart Center Bonn in 2017 (n = 2484) were screened for high-pressure dilatation. For this purpose, all cardiac PCI catheterization protocols were reviewed and those patients who were treated with an NC balloon and a minimum dilatation pressure of ≥ 16 atmospheres (atm) were deemed eligible out (n = 963). In a further step, the images of the coronary angiograms were examined for moderate and severe calcification. Those patients in whom high-pressure dilatation was performed as a therapy for a calcified stenosis (n = 389) were selected for propensity-score-matched (PSM) analysis. Patients undergoing bypass graft intervention and patients who had a cardiac arrest before/during the intervention were excluded from this study.

The calcified lesion was angiographically graded as either moderate or severe. Moderate calcification was defined as radiopacities noted only during the cardiac cycle before the injection of contrast dye. Severe calcification was defined as radiopacities seen without cardiac motion before the injection of contrast dye.

A systematic follow‐up of all patients was conducted at 12-months by clinical visits or telephone calls. Data were collected by reviewing medical records and follow- up was performed by a telephone interview of the patients or the general practitioner.

The local institutional ethics committee approved this registry. All patients signed an informed consent form before the follow-up data were recorded.

### 2.2 Intravascular lithotripsy

We used the Shockwave C^2^ (Shockwave Medical Inc., California, USA) balloon-based coronary IVL system. The Shockwave balloon device was prepared according to the manufacturer’s instructions for use and positioned at the target lesion. A 1:1 ratio of Shockwave balloon diameter to planned stent diameter was chosen. In cases where the Shockwave balloon was not deliverable in the lesion, we performed a predilatation with a semi-compliant balloon.

The balloon catheter was inflated to 4 atm and up to 10 sonic pressure impulses were delivered at a time. The balloon was then inflated to 6 atm and then deflated to reestablish blood flow. In total, up to 80 impulses were delivered, with the possibility of balloon relocation within the lesion. Each treatment of 10 impulses was terminated with a 6 atm inflation of the Shockwave balloon.

### 2.3 End points

Primary end point of this study was procedural success, defined as successful stent delivery and expansion, with the attainment of < 20% residual in-stent stenosis of the target lesion in the presence of TIMI 3 (thrombolysis in myocardial infarction) flow without stent failure. The safety outcome was procedural complications, defined as coronary dissection, slow or no reflow, new coronary thrombus formation during PCI, abrupt vessel closure and device failure (inability to place the balloon, malfunction, or burst), and in-hospital major adverse cardiovascular event (MACE), which was defined as proposed by the American Heart Association and the Academic Research Consortium-2 in the fourth universal definition for myocardial infarction associated with PCI.

Secondary end points included death, stroke, myocardial infarction, stent thrombosis, target-lesion revascularization, target-vessel revascularization, and any revascularization.

### 2.4 Quantitative coronary angiography

Baseline, postprocedural, and final coronary angiograms were digitally recorded, and quantitative coronary angiography was performed offline. Measurements were performed using the same single worst-view projection. A contrast-filled non-tapered catheter tip was used for calibration.

### 2.5 In-hospital follow-up

For postinterventional antiplatelet therapy, all patients received 100 mg of aspirin per day. Additionally, patients with chronic coronary syndrome received 75 mg of clopidogrel for at least six months, and patients with acute coronary syndrome received ticagrelor 180 mg/d. Patients who taking oral anticoagulants were treated with a dual therapy, combined with clopidogrel, for at least six months. During their hospital stay, patients were clinically monitored for the occurrence of any adverse events and any additional coronary interventional treatment.

### 2.6 12-month follow-up

12-month follow-up data were collected during routine out of patient visits in our department.

### 2.7 Statistical analysis

Baseline and procedural characteristics were summarized for the overall cohort. Categorical variables were presented as counts and percentages and were compared using a χ^2^ test or Fisher exact test, as required. Continuous variables were presented as the mean ± standard deviation or median (interquartile range) and were compared using a 2-sided unpaired *t* test or a Mann–Whitney *U* test, as appropriate. A 2-sided p value < 0.05 was considered statistically significant.

We applied a method for propensity-score matching (PSM) to adjust for confounding baseline variables. PSM was modelled using a multivariate logistic regression model based on the following baseline characteristics: age, gender, dyslipidemia, smoking, prior history of AMI and PCI, history of stroke and chronic kidney disease, clinical presentation due to acute coronary syndrome including ST elevation myocardial infarction (STEMI) and non-ST elevation myocardial infarction (NSTEMI), lesion localization, lesion length, and lesion assessment. Matching was performed without replacement and with a caliper of 0.2 of the standard deviation of the logit of the propensity score. PSM was performed by using *R project for statistical computing* (Vienna, Austria).

## 3 Results

### 3.1 Propensity-score matching and baseline characteristics

**Fig. 1:**
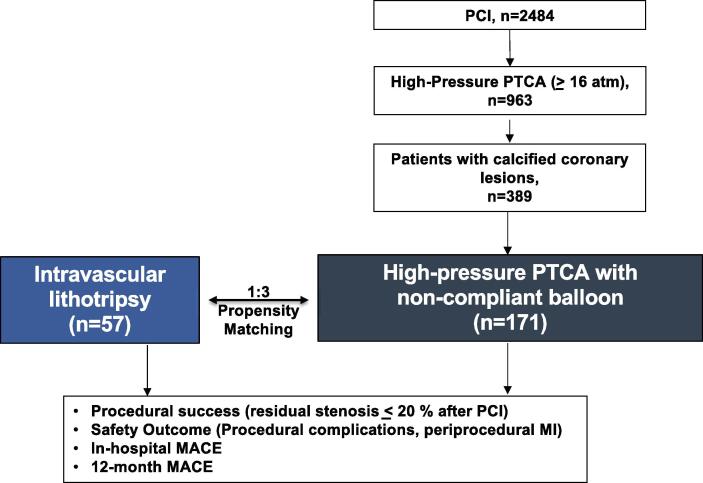
**Study flow chart.** The comparison groups consist of a cohort of patients with calcified coronary lesions who were treated with IVL, or high-pressure non-compliant (NC) balloon dilatation. All patients who underwent PCI were screened for high-pressure dilatation. To this end, all cardiac catheter protocols were reviewed and those patients who were treated with an NC balloon and a minimum dilatation pressure of ≥ 16 atmospheres (atm) were filtered out (n = 963). Next, the images of the coronary angiograms were examined for moderate and severe calcification. Those patients in whom high-pressure dilatation was performed as a therapy for a calcified stenosis (n = 389) were selected for propensity-score-matched (PSM) analysis and compared with IVL guided PTCA.

A total of 228 patients were included in this study. The study flowchart is depicted in Fig. 1. Propensity-score matching (PSM) was performed in a 3:1 manner and resulted in 171 matched pairs. Baseline characteristics of the study patients are displayed in Table 1. Baseline and lesion characteristics of the unmatched high-pressure group are displayed in Online Table 1, Table 2. After matching for the covariates, there were no significant differences in baseline characteristics with respect to cardiovascular risk factors, history of coronary interventions, prevalence of chronic renal failure, or clinical presentation (mostly chronic coronary syndrome) between the IVL group (n = 57) and the matched standard high-pressure group (n = 171). The mean age of the IVL group was 75.9 ± 9.9 years and 74.7 ± 11.9 years in high-pressure group. Prevalence of male sex was high in both groups (73.7% vs. 75.4%) as was the distribution of cardiovascular risk factors (hypertension 91.2% vs. 87.1%, dyslipidemia 64.9% vs. 55.6%, diabetes mellitus 35.1% vs 36.3%, previous, current, or former smoking 31.6% vs. 32.2%). Prevalence of chronic kidney disease was 35.1% in the IVL group vs. 29.8% in the high-pressure group.

**Table 1:** Baseline characteristics.

	**IVL**	**High-pressure PTCA**	***p* value**	
			***matched***	***unmatched***
**Number of patients**	57	171	–	
Male (n, %)	42 (73.7)	129 (75.4)	0.79	0.75
Age (mean ± SD)	75.9 (±9.9)	74.7 (±11.9)	0.48	0.5
**Cardiovascular risk factors/ Comorbidities**				
Hypertension (n, %)	52 (91.2)	149 (87.1)	0.41	0.06
Dyslipidemia (n, %)	37 (64.9)	95 (55.6)	0.21	**<0.0001**
Smoking (n, %)	18 (31.6)	55 (32.2)	0.93	**0.02**
Family history of CVD (n, %)	4 (7.2)	14 (8.2)	0.89	0.24
Diabetes (n, %)	20 (35.1)	62 (36.3)	0.87	0.89
Adipositas (n, %)	15 (26.3)	44 (25.7)	0.93	0.66
Prior MI (n, %)	30 (52.6)	69 (40.4)	0.10	**<0.0001**
Prior CABG (n, %)	7 (12.3)	16 (9.4)	0.52	0.22
Prior PCI (n, %)	31 (54.4)	89 (52.1)	0.76	**0.04**
Atrial fibrillation (n, %)	20 (35.1)	64 (37.4)	0.75	0.29
LVEF (mean ± SD %)	51.24(±13.58)	52.04(±12.97)	0.70	0.40
Stroke (n, %)	13 (22.9)	23 (13.5)	0.09	**<0.0001**
CKD* (n, %)	20 (35.1)	51 (29.8)	0.45	**0.01**
Creatinine (mean ± SD; mg/dl)	1.27 (±0.82)	1.61 (±1.4)	0.11	0.19
PAD (n, %)	16 (28.1)	35 (20.5)	0.23	0.06
COPD (n, %)	10 (17.5)	18 (10.5)	0.16	0.2
**Clinical presentation**				
ACS (n, %)	16 (28.1)	61 (35.7)	0.29	**0.02**

Values are n (%) or mean ± SD.

IVL: intravascular lithotripsy; PTCA: percutaneous transluminal coronary angioplasty; CVD: cardiovascular disease; MI: myocardial infarction; CABG: coronary artery bypass graft; LVEF: left ventricular ejection fraction; PCI: percutaneous coronary intervention; CKD: chronic kidney disease; PAD: peripheral artery disease; COPD: chronic obstructive pulmonary disease; ACS: acute coronary syndrome. *Defined as a glomerular filtration rate < 60 mL/min.

**Table 2:** Lesion and procedural characteristics.

**Target vessel**	**IVL**	**High-pressure PTCA**	***p* value**	
			***matched***	***unmatched***
Left main (n, %)	9 (15.8)	29 (16.7)	0.83	0.26
LAD (n, %)	26 (45.6)	78 (45.6)	0.99	**0.04**
RCX (n, %)	3 (5.3)	20 (11.7)	0.16	0.06
RCA (n, %)	19 (33.3)	45 (26.3)	0.3	0.65
**Lesion localization**				
Proximal (n, %)	28 (49.1)	82 (47.9)	0.88	0.42
Medial (n, %)	20 (35.1)	63 (36.8)	0.81	**<0.0001**
Distal (n, %)	9 (15.8)	26 (15.2)	0.92	0.47
**Lesion characteristics**				
Length (mean ± SD, mm)	10.08 (±9.45)	10.23 (±10.13)	0.88	0.90
**Calcification**				
Moderate (n, %)	10 (17.5)	33 (19.3)	0.76	0.08
Severe (n, %)	47 (82.5)	138 (80.7)	0.77	0.08
Eccentric (n, %)	34 (59.7)	101 (24)	0.93	**<0.0001**
Concentric (n, %)	23 (40.3)	70 (76)	0.93	**<0.0001**
**Procedural characteristics**				
Vascular access				
Femoral artery	38 (66.7)	101 (59.1)	0.30	0.77
Radial artery	19 (33.3)	70 (40.9)	0.31	0.47

Values are n (%) or mean ± SD.

IVL: intravascular lithotripsy; PTCA: percutaneous coronary angioplasty; LAD: left anterior descending coronary artery; RCX: ramus circumflexus; RCA: right coronary artery.

### 3.2 Lesion and procedural characteristics

Lesion characteristics and procedural characteristics are shown in Table 2. The anterior descending artery (LAD) was the target vessel in 45.6% of cases in the IVL group and in 45.6% of cases in the high-pressure group. The right coronary artery (RCA) was treated in 33.3% of cases in the IVL group and in 26.3% of cases in the high-pressure group. Additionally, the left main vessel was the target in 15.8% of cases in the IVL group and in 16.7% of cases in the high-pressure group. Out of 57 patients in the IVL group, 47 (82.5%) were severely calcified, while 10 lesions (17.5%) were considered moderately calcified. 138 patients (80.7 %) in the high-pressure group had severely calcified lesions, whereas 33 patients (19.3%) had moderately calcified lesions.

Procedural details for IVL are shown in Table 3. The average number of pulses applied was 66 ± 27. The mean diameter of the lithotripsy balloon catheter was 3.38 ± 0.37 mm. In 19 of 57 cases (33.3%), post-IVL high-pressure NC-balloon PTCA with a mean pressure of 29.2 ± 7.8 atm was necessary. Post-stent NC-balloon dilatation was performed in 33.3% of cases.

**Table 3:** Intravascular lithotripsy procedural characteristics.

	**IVL (n = 57)**
Amount of pulses applied (mean ± SD, n)	66 ± 27
Diameter of lithotripsy balloon, (mean ± SD, mm)	3.38 ± 0.37
Post IVL high-pressure dilatation (n, %)	19 (33.33)
Diameter of post-dilatation balloon (mm, mean ± SD)	3.25 ± 1.02
Mean pressure (atm ± SD)	29.2 ± 7.8
Post-stent dilatation (n, %)	19 (33.3)
Diameter of post-stent dilatation balloon (mean ± SD, mm)	3.8 ± 1.1
Mean pressure (atm, mean ± SD)	17.3 ± 5.6
Values are n (%) or mean ± SD.	

IVL: intravascular lithotripsy. atm: atmosphere.

Procedural details for high-pressure PTCA are displayed in Table 4. The mean diameter of the high-pressure NC-balloon was 2.87 ± 1.0 mm, and the mean pressure was 20.1 ± 4.0 atm. Post-stent NC-balloon dilatation was needed in 45.0% of cases.

**Table 4:** High pressure dilatation procedural characteristics

	**High-Pressure PTCA (n = 171)**
Diameter of high-pressure balloon, (mean ± SD, mm)	3.0 (±1.0)
Mean pressure of dilatation (mean ± SD, atm)	20.1 (±4.0)
Post-stent dilatation (n, %)	77 (45.0)
Diameter of post-stent dilatation balloon (mean ± SD, mm)	3.5 (±1.77)
Mean pressure (atm, mean ± SD)	16.63 (±2.95)
Values are n (%) or mean ± SD. atm: atmosphere.	

### 3.3 Clinical outcomes

**Fig. 2:**
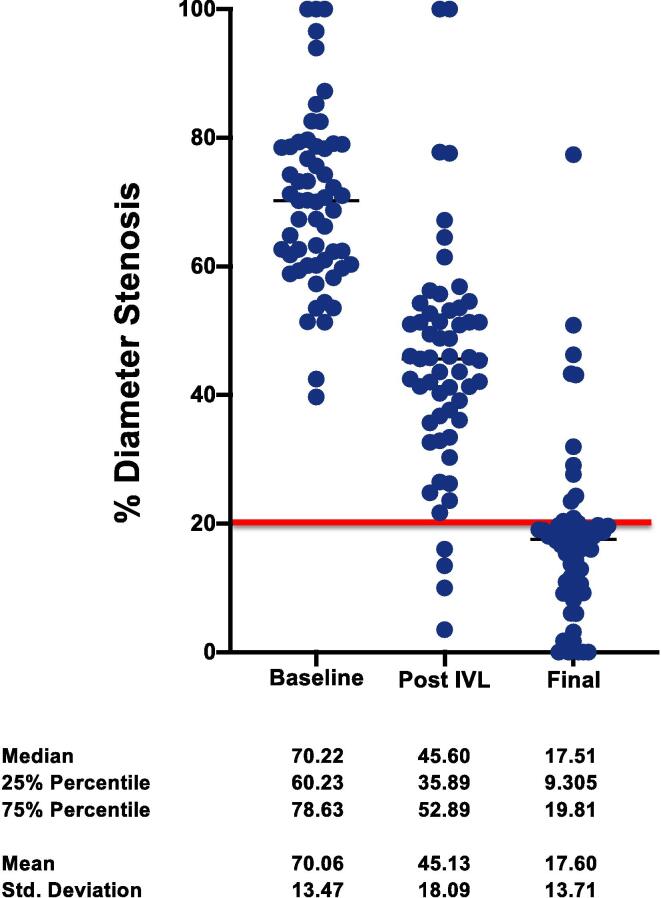
**Quantitative Coronary Analysis (QCA) showing % diameter stenosis for IVL group.** The red line indicates the clinically relevant residual stent stenosis after PCI.

**Fig. 3:**
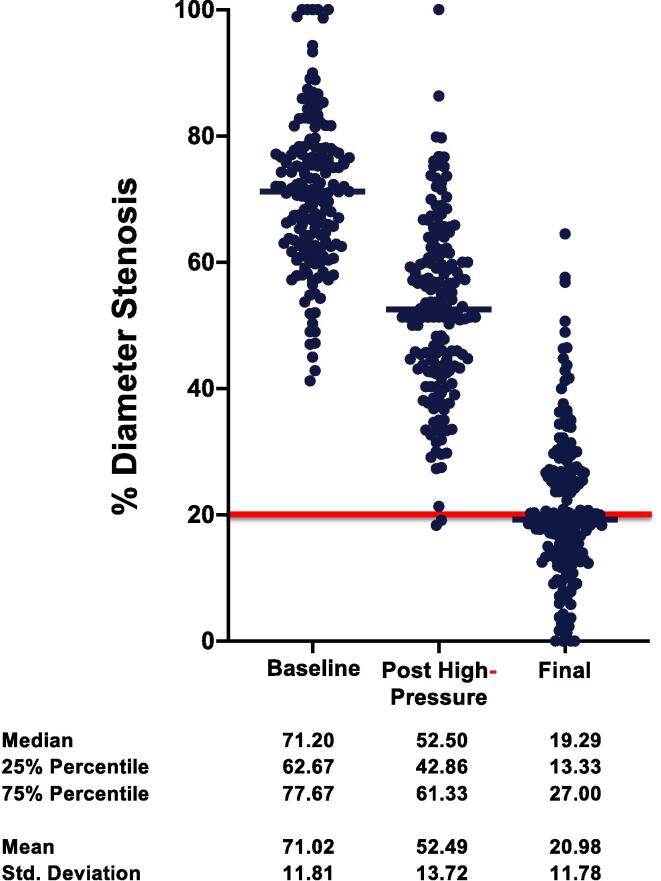
**Quantitative Coronary Analysis (QCA) showing % diameter stenosis for high-pressure group.** The red line indicates the clinically relevant residual stent stenosis after PCI.

The results of quantitative coronary angiography are demonstrated in Table 5, and Fig. 2, Fig. 3. In the IVL group, MLD was 1.08 ± 0.51 mm at baseline with a median diameter stenosis of 70.2% (interquartile range, 60.2–78.6). After lesion preparation, with IVL, stenosis was reduced to 45.6% (interquartile range, 35.9–52.9), with a mean acute gain of 0.93 ± 0.7 mm. After DES implantation, residual stenosis was 17.5% (interquartile range, 9.3–19.8) with a mean acute gain of 1.86 ± 0.61 mm to baseline (Table 5, Fig. 2). In high-pressure- PTCA group, MLD was 0.97 ± 0.43 mm at baseline with a median diameter stenosis of 71.2 % (interquartile range, 62.67–77.67). After lesion preparation with high- pressure PTCA stenosis was reduced to 52.5 % (interquartile range, 42.86–61.33) with a mean acute gain of 0.6 ± 0.47 mm. After drug-eluting stent (DES) implantation, residual stenosis was 19.29% (interquartile range, 13.33–27.00), with a mean acute gain of 1.62 ± 0.5 mm to baseline (Table 5, Fig. 3).

**Table 5:** Quantitative Coronary Analysis.

	**IVL**			**High-Pressure PTCA**		
	**Baseline**	**Post IVL**	**Post PCI**	**Baseline**	**Post High-Pressure**	**Post PCI**
**RVD**	3.55 ± 0.48	3.31 ± 0.55				
**MLD**	1.08 ± 0.51	1.9 ± 0.63	2.91 ± 0.56	0.97 ± 0.43	1.58 ± 0.48	2.6 ± 0.47
**Acute gain**	–	0.93 ± 0.7	1.86 ± 0.61	–	0.6 ± 0.47	1.62 ± 0.5
**% DS**	70.1 ± 13.5	45.13 ± 18.1	17.6 ± 13.7	71.02 ± 11.81	52.49 ± 13.72	20.98 ± 11.78

Values are mean ± SD.

IVL, intravascular lithotripsy; PTCA: percutaneous translumimal coronary angioplasty; RVD: reference vessel diameter; MLD: minimal lumen diameter; DS: diameter stenosis; PCI, percutaneous coronary intervention.

**Fig. 4:**
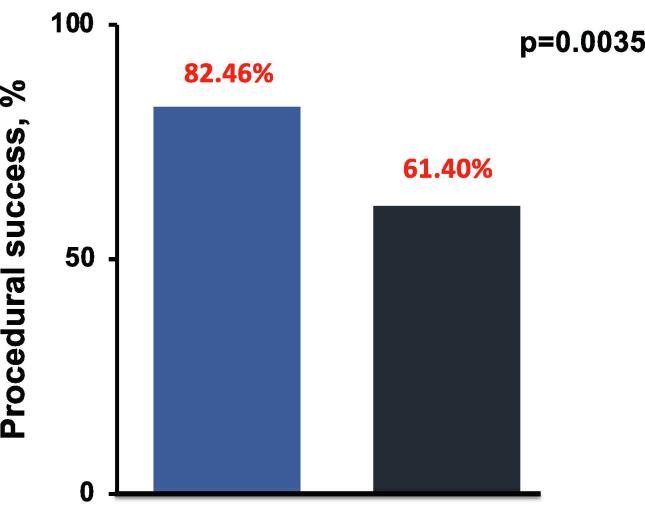
**Procedural success for IVL and high-pressure group. Procedural success** was the primary endpoint with success rates of 82.46% in IVL group, and 61.40% in high-pressure group.

Primary endpoints are shown in Table 6. Procedural success was reached in 82.5% of patients in the IVL group, and 61.4% of patients in the high-pressure PTCA group (p = 0.035, Fig. 4). Device failure (burst, rupture) occurred more often in the IVL group (12.3% vs. 1.2%, p=<0.0001). None of these events led to further complications (e.g., vessel rupture/pericardial effusion).

**Table 6:** Primary endpoints: procedural success and safety outcomes.

	**IVL (n = 57)**	**High-Pressure PTCA (n = 171)**	***p* value**
**Procedural success**			
Successful stent delivery and expansion with < 20% residual stenosis of the target lesion, TIMI 3 flow, no stent failure	47 (82.5)	105 (61.4)	**0.0035**
**Safety outcome**			
Device failure (burst, rupture),	7 (12.3)	2 (1.2)	**<0.0001**
In-hospital MACE (MI, TVF, or cardiac death)	0	0	–
Complete lithotripsy treatment at target lesion	57 (1 0 0)	–	–
Coronary dissection	0 (0)	0 (0)	–
Perforation	0 (0)	0 (0)	–
Slow-flow phenomenon	0 (0)	0 (0)	–
No-reflow phenomenon	0 (0)	0 (0)	–
Abrupt vessel closure	0 (0)	0 (0)	–

Values are n (%) or mean ± SD.

IVL, intravascular lithotripsy; PTCA: percutaneous translumimal coronary angioplasty; TIMI: thrombolysis in myocardial infarction; MACE: major adverse cardiac event; MI: myocardial infarction; TVF: target vessel failure.

Baseline and lesion characteristics of patients with procedural success, and patients with procedural failure in the IVL group are summarized in Online Table 3, Table 4 in the data supplement. No differences in baseline or lesion characteristics, procedural, or IVL characteristics could be identified (Online Table 3, Table 4, Table 5, Table 6 in the data supplement).

**Fig. 5:**
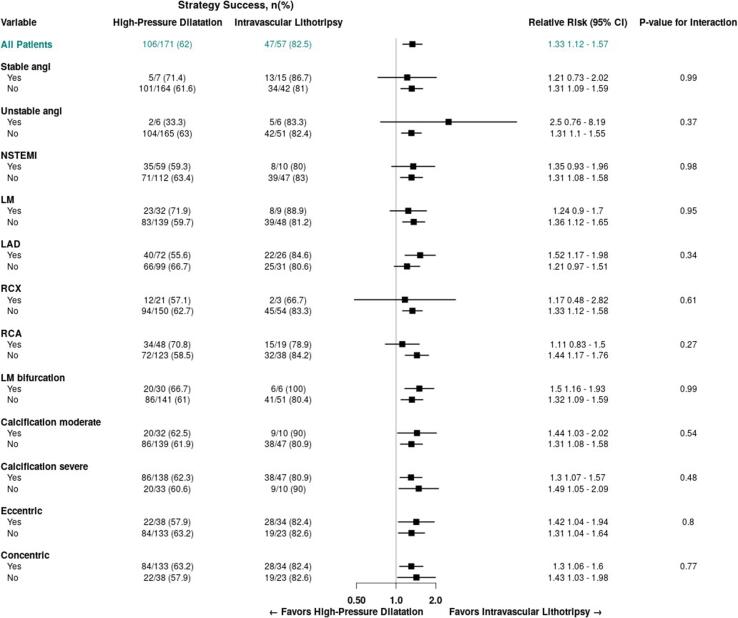
**Subgroup analyses for procedural success.** Subgroup analyses are shown for the primary end point of procedural success among patients who were assigned to undergo lesion preparation with either a modified balloon or rotational atherectomy. The p value for interaction represents the likelihood of interaction between the variable and the relative treatment effect. Risk ratios are for IVL versus high-pressure PTCA.

Results, with respect to the primary end point, were consistent in prespecified subgroups (Fig. 5).

### 3.4 In-hospital outcomes

There was no in-hospital MACE in any of the patients in both groups. One patient (2.5%) from the IVL group suffered from unstable angina seven days after the procedure. Invasive diagnostics showed no signs of target or non-target lesion failure.

### 3.5 12-Month clinical outcome

Complete clinical follow-up over 12 months was available for all 228 patients (100%). MACE through 12 months occurred in 10.5% of cases in the IVL group and 11.1% of cases in the high-pressure group (p = 0.22). Overall mortality was 5.2% in the IVL group, and 6.4% in the high-pressure group (p = 0.79). Between discharge and 12 months, myocardial infarction occurred in 3 patients (5.2%) in the IVL group and 8 patients (4.7%) in the high-pressure group. Clinically indicated, target-vessel revascularization was similar in both groups, with low event rates and no statistically significant difference (1.8% vs 1.1%; p = 0.50). There were no definitive cases of stent thrombosis observed in either group (Table 7).

**Table 7:** Twelve-month clinical outcome (n = 228 Patients)

	**IVL** **(n = 57)**	**High-Pressure** **PTCA** **(n = 171)**	***p* value**
**Death**	3 (5.2%)	11 (6.4%)	0.79
Cardiac death	2 (3.5%)	7 (4.1%)	0.64
Noncardiac death	1 (1.8%)	4 (2.3%)	0.10
**MI**	3 (5.2%)	8 (4.7%)	0.12
Target vessel MI	1 (1.8%)	2 (1.2%)	0.45
TVR	1 (1.8%)	2 (1.2%)	0.45
Stent thrombosis (definite)	0 (0%)	0 (0%)	–
TLR	0 (0%)	0 (0%)	–
Any revascularization	11 (19.3%)	23 (13.5)	0.17

Values are n (%).

IVL, intravascular lithotripsy; PTCA: percutaneous translumimal coronary angioplasty; MI: myocardial infarction; TVF: target vessel revascularization; TLR: target lesion revascularization.

## 4 Discussion

To the best of our knowledge, this study is the first comparing primary IVL with a standard strategy for lesion preparation in patients with calcified coronary lesions. In an elderly population with complex calcified coronary artery disease, the procedural success rate was higher with primary IVL. Safety was similar in both groups. In-hospital follow-up and the 12-month outcome were not significantly different.

This retrospective study was designed to compare the safety and efficacy of primary IVL prior to coronary DES implantation using standard procedures for lesion preparation in calcified lesions. Lesion preparation beginning with high-pressure dilatation is standard in most catheterization laboratories. In our cohort of the high-pressure dilatation group, the endpoint of procedural success was met only in 61.4 % of cases, which was surprisingly low. These results are likely due to the retrospective nature of the study. Nevertheless, this represents the clinical routine and illustrates the need for more adequate strategies of lesion preparation prior to stenting. Residual in-stent stenosis of < 20% might be a tough endpoint to achieve, but it is associated with a better clinical outcome. For analyses of the patients with an unsuccessful procedure (those with residual in-stent stenosis > 20%), preprocedural intravascular imaging would have improved the analysis of the failures [16], [17], [18].

Studies, particularly randomized trials, comparing devices in calcified lesions are scarce. The enrollment criteria for our study were similar to the only randomized trials performed so far: ROTAXUS (Rotational Atherectomy Prior to Taxus Stent Treatment for Complex Native Coronary Artery Disease) and PREPARE-CALC (Comparison of Strategies to Prepare Severely Calcified Coronary Lesions) [19], [20]. Considering the baseline characteristics of the cohorts and endpoints of procedural success of the ROTAXUS and PREPARE-CALC studies, primary IVL was less likely than rotational atherectomy to meet the efficacy endpoint, however rotational atherectomy was shown to be superior for procedural success, when compared to standard therapy and modified balloons (cutting/scoring balloons). This result may be because of the following reasons: First of all, ROTAXUS and PREPARE-CALC excluded patients with acute or recent myocardial infarction, which are known to lead to unfavorable procedural and clinical outcomes. In our study, acute coronary syndrome was the reason for clinical presentation in 28.1% and 35.7% of cases, respectively. Secondly, cohorts of both of these trials had fewer patients with chronic renal failure (ROTAXUS: 4.7%, 6.7% of cases, respectively, and PREPARE-CALC: 21%, 26% of cases, respectively). Moreover, the patients in PREPARE-CALC were much younger than in our cohorts. These points might also be the reason why the standard therapy of our cohort has a significantly worse procedural result compared to standard therapy in ROTAXUS and modified balloons in PREPARE-CALC. The MACE rate through 12 months was, in our analyses, higher compared to the ROTAXUS and PREPARE-CALC cohorts. Registry data from a non-randomized, single-arm ORBIT II Trial (Pivotal Trial to Evaluate the Safety and Efficacy of the Orbital Atherectomy Systemin Treating De Novo, Severely Calcified Coronary Lesions) showed a success rate of 88.9%[21]. However, their definition of success differs blatantly from our study as well as from ROTAXUS and PREPARE-CALC. In the ORBIT II trial, efficacy was defined as residual stenosis < 50% post-stent without in-hospital major adverse cardiac events. This might explain the high rate of MACE (15.1%), mainly driven by TVR (5.2%), and TLR (3.9%), in a group of patients treated with 2nd generation DES [22].

In this study, device failure occurred more often in the IVL group (12.3% vs. 1.2%), in all cases driven by balloon rupture. Balloon rupture during PCI is not common. But is has been reported to be associated more frequently in the presence of calcified lesions. Although balloon rupture is not considered a significant problem during PCI, serious complications can occur, such as dissection, coronary perforation, and air embolism. Case reports have described IVL balloon rupture during lithotripsy with important vessel dissection [23], [24]. The reasons here fore are not well understood. Balloon rupture is likely due to the morphology of the lesion, the presence of critical stenosis and the presence of severe vessel tortuosity [25], [26], [27]. However, data of systematic investigation of balloon rupture in calcified lesions are lacking.

This propensity matched study provides new data, that confirm the efficacy and safety of IVL, and showed superiority in procedural success with lesion preparation compared to the standard strategy. Furthermore, the MACE rate of our patients through 12 months after IVL was similar to the standard therapy, despite higher rates of initial angiographic success with IVL.

However, randomized trials comparing high-pressure NC-dilatation, rotational atherectomy, and IVL are still required to define the relative safety and effectiveness of these devices. Furthermore, such data would help determine if device performance is dependent on the type of lesion presentation.

### 4.1 Limitations

Our study has some limitations: 1) This is a single-center, retrospective study. A randomized study comparing IVL against conventional noncompliant balloon dilation or scoring/cutting balloon strategies would improve our knowledge of the efficacy and safety of the technique. 2) Patient inclusion into the study was based on the angiographic degree of calcification and not on intravascular imaging. Optical coherence tomography/intravascular ultrasound were done in ≈25% of cases. This represents well the clinical routine in an all-comers cohort. However, for analyses of patients with an unsuccessful procedure (those with residual in-stent stenosis > 20%), preprocedural intravascular imaging would have improved our failure analysis. 3) IVL may have limitations in asymmetrical calcifications. These clinical situations, as well as cost analyses, have not yet been performed.

## 5 Conclusion

IVL resulted in a significantly higher rate of procedural success, compared to high-pressure NC-balloon dilatation in patients with calcified coronary lesions. Despite the high rates of initial angiographic success using IVL, the MACE rate through 12 months was similar to standard therapy.

## Funding

This work was supported by the Clinical Study Research program (IUT) of the University Hospital Bonn.

## Declaration of Competing Interest

The authors declare that they have no known competing financial interests or personal relationships that could have appeared to influence the work reported in this paper.
